# Co‐culture of human fibroblasts, smooth muscle and endothelial cells promotes osteopontin induction in hypoxia

**DOI:** 10.1111/jcmm.14905

**Published:** 2020-02-07

**Authors:** Nirvana Sadaghianloo, Julie Contenti, Maeva Dufies, Julien Parola, Matthieu Rouleau, Shinrong Lee, Jean‐François Peyron, Lucilla Fabbri, Réda Hassen‐Khodja, Jacques Pouysségur, Frédéric Bost, Elixène Jean‐Baptiste, Alan Dardik, Nathalie M. Mazure

**Affiliations:** ^1^ Université Côte d’Azur Institute for Research on Cancer and Aging of Nice (IRCAN) CNRS‐UMR 7284‐Inserm U1081 Centre Antoine Lacassagne University of Nice Sophia‐Antipolis Nice France; ^2^ Department of Vascular Surgery Centre Hospitalier Universitaire de Nice Nice France; ^3^ Department of Emergency Medicine Centre Hospitalier Universitaire de Nice Nice France; ^4^ Centre Scientifique de Monaco (CSM) Monaco Monaco; ^5^ Faculty of Medicine LP2M CNRS‐UMR 7370 Nice France; ^6^ Department of Surgery and the Vascular Biology and Therapeutics Program Yale University New Haven CT USA; ^7^ Department of Vascular Surgery VA Connecticut Healthcare Systems West Haven CT USA; ^8^ Present address: Centre de Méditerranéen de Médecine Moléculaire (C3M) INSERM U1065 Université Côte d’Azur Nice Cedex 03 France

**Keywords:** arteriovenous fistula, hypoxia, hypoxia‐inducible factor, metabolism, osteopontin

## Abstract

Arteriovenous fistulas (AVFs) are the preferred vascular access for haemodialysis of patients suffering from end‐stage renal disease, a worldwide public health problem. However, they are prone to a high rate of failure due to neointimal hyperplasia and stenosis. This study aimed to determine if osteopontin (OPN) was induced in hypoxia and if OPN could be responsible for driving AVF failure. Identification of new factors that participate in remodelling of AVFs is a challenge. Three cell lines representing the cells of the three layers of the walls of arteries and veins, fibroblasts, smooth muscle cells and endothelial cells, were tested in mono‐ and co‐culture in vitro for OPN expression and secretion in normoxia compared to hypoxia after silencing the hypoxia‐inducible factors (HIF‐1α, HIF‐2α and HIF‐1/2α) with siRNA or after treatment with an inhibitor of NF‐kB. None of the cells in mono‐culture showed OPN induction in hypoxia, whereas cells in co‐culture secreted OPN in hypoxia. The changes in oxygenation that occur during AVF maturation up‐regulate secretion of OPN through cell‐cell interactions between the different cell layers that form AVF, and in turn, these promote endothelial cell proliferation and could participate in neointimal hyperplasia.

## 1 INTRODUCTION

Renal failure is a major public health problem with increasing incidence every year. Patients with end‐stage renal disease require either kidney transplantation or haemodialysis to sustain life. Arteriovenous fistula (AVF) is the preferred vascular access for haemodialysis.^1^ However, only 50% of AVF remain functional six months after creation, which increases the morbidity‐mortality of patients.^2^ Several clinical factors seem to play a role in the dysfunction of AVFs, including female gender, age, or diabetes. Histologically, dysfunction most frequently results from neointimal hyperplasia (NH), which is responsible for stenosis.^3^ Different mechanisms including the differentiation of fibroblasts into myofibroblasts, proliferation of smooth muscle cells and endothelial cell activation have been identified in NH of AVF. Following surgery, changes in tissue oxygenation result in (a) an increase of arterial O_2_ partial pressure (pO_2_), from 35 to 45 mm Hg in the venous blood and from 73 to 100 mm Hg in the arterial blood, and (b) a decrease in the oxygen (O_2_) concentration in the venous wall, as seen by the stabilization of the hypoxia‐inducible factor (HIF) in the venous limb of the AVF in several models.^4^ Rupture of the *vasa vasorum* during surgical dissection may contribute to the induction of hypoxia, thereby stabilizing HIF.^5^ HIFs are dimeric protein complexes that consist of an α‐subunit (HIF‐1α or HIF‐2α) and a β‐subunit (HIF‐1β or HIF‐2β),^6^ and are major regulators of cellular adaptation to hypoxia. HIF‐1α is expressed ubiquitously, whereas HIF‐2α is primarily detected in endothelial cells but is also selectively highly expressed in a limited number of tissues.^7^ There is increasing evidence supporting the contribution of the HIF pathway, both the protective and destructive effects, to the pathogenesis of diseases affecting the vascular wall including atherosclerosis,^8^, ^9^ arterial aneurysms,^10^, ^11^, ^12^ pulmonary hypertension,^13^, ^14^, ^15^ vascular graft failure,^4^, ^16^, ^17^, ^18^ chronic venous diseases^19^, ^20^ and vascular malformation.^21^, ^22^ Furthermore, increased expression of *VEGF‐A*, a target HIF‐1α gene, may contribute to NH, through increased proliferation of smooth muscle cells. A recent study has shown that reducing *VEGF‐A* gene expression during AVF formation reduces NH.^23^

Although HIFs are involved in the regulation of the oxygen homeostasis, NF‐κB, a major transcription factor that responds to cellular stress, is also activated by hypoxia.^24^ The most abundant cytoplasmic form of the NF‐κB complex is an inactive heterotrimeric form composed of p50 and p65 subunits, and the inhibitor IKB‐α. Stimulus‐induced degradation of IKB‐α is critical for nuclear translocation of NF‐κB and induction of transcription of target genes.^25^ In rat models, overexpression of NF‐κB was found following vascular injury and correlated to thickening of the intima compared to that of control vessels.^26^

Osteopontin (OPN) is a SIBLING protein (Small Integrin Binding Ligand N‐linked Glycoproteins), which was initially identified as a bone matrix protein that links bone cells to the extracellular matrix.^27^ OPN exists in two isoforms, a secreted (sOPN) and an intracellular form (iOPN), that have distinct biological functions.^28^ At the protein level, OPN has a molecular weight of about 60 kD. This protein undergoes multiple post‐translational modifications by phosphorylation and glycosylation variables that can explain the previously described variability in the apparent molecular weights (from 25 to 75 kD).^29^ OPN is involved in multiple processes including tissue remodelling, regulation of cellular immunity, pathological chronic inflammatory processes, carcinogenesis as well as cardiovascular diseases.^30^ OPN is also involved in several vascular diseases promoting angiogenesis, in parallel with vascular endothelial growth factor (VEGF), through enhanced endothelial cell migration, proliferation and subsequent formation of capillaries, which are essential requirements for the process of angiogenesis. In particular, it has been found to be expressed in vascular smooth muscle cells of human restenotic lesions and stenotic vascular lesions.^31^ Significant association between the level of plasma circulating OPN and atheroma plaque formation has been reported.^32^ Moreover, high OPN levels in patients with stenosis have been described after coronary angioplasty compared to patients without stenosis.^33^ Interestingly, Hall et al^34^ have shown a 40‐fold increase in OPN expression in the early stages of AVF maturation in a mouse model of AVF. In addition, constitutive overexpression of OPN in mice was found to result in increased neointima formation after cuffing of the femoral artery.^35^ Finally, structural changes were noted on the OPN‐null background including disorganized collagen and increased vessel wall compliance.^36^ Altogether, these data suggest that OPN may play a role in the development of vascular stenosis associated with excessive intimal proliferation. Reactive oxygen species (ROS)^37^ and hyperglycaemia^38^ induce in vivo expression of OPN in pancreatic epithelial cells, and also in vascular smooth muscle cells. Even though up‐regulation of OPN in hypoxia has been shown to be dependent^39^, ^40^ or independent^41^ of HIF‐1 regulation, OPN expression is enhanced under hypoxia through different mechanisms,^42^ leading to co‐expression with VEGF.^43^ Moreover, we reported that the oxidative stress generated during early maturation of an AVF stabilized the HIF‐1α protein and thus activated HIF‐target genes such as *Vegf‐A*, *Nox‐2* and *Ho‐1*.^44^

Given that changes in the oxygen concentration occur during surgical creation of an AVF in parallel to OPN overexpression, we suggested that OPN is induced by hypoxia and thus could contribute to the failure of AVF maturation due to NH and juxtaanastomotic stenosis. We postulated that regulation of OPN through silencing of HIFs or NF‐kB could promote AVF maturation. Finally, we describe an all‐human *in vitro* cell model of co‐culture that induces OPN in hypoxia but only under conditions of cell‐cell interaction.

## 2 MATERIALS AND METHODS

### 2.1 Cell culture and co‐culture

Normal human fibroblasts (NHF; gifted by Dr Cédric Gaggioli, IRCAN) were grown in Dulbecco's modified eagle medium (DMEM, Gibco) supplemented with 10% foetal bovine serum (FBS), penicillin (10 U/mL) and streptomycin (10 μg/mL), and used between passages 3 and 10. Human umbilical vein smooth muscle cells (HUVSMC) were grown on poly‐L‐lysine‐coated dishes in full smooth muscle sell medium (ScienCell Research Laboratories) and used between passages 1‐6. Human umbilical vein endothelial cells (HUVEC; Gibco, Thermo Fisher Scientific) were grown on gelatin‐coated dishes in endothelial cell growth medium supplemented with SingleQuots^TM^ (Lonza), penicillin (10 U/mL) and streptomycin (10 μg/mL), and used between passages 1 and 6. Mesenchymal stromal cells (MSCs) were isolated from bone marrow aspirate obtained from healthy donors and expanded in alpha‐MEM medium (Gibco; Thermo Fisher Scientific) supplemented with 5% HyClone foetal bovine serum (Thermo Fisher Scientific) while fulfilling uniformly minimal MSC criteria. Their osteoblast differentiation was obtained after 3 weeks of culture in the StemPro Osteogenesis Differentiation kit (Thermo Fisher Scientific) according to the manufacturer's instructions. For normoxia, cells were maintained in a humid incubator at 37°C with 5% CO_2_ and air. For hypoxia, cells were transferred to an *Invivo_2_* hypoxic workstation (Baker Ruskinn Global) set at 37°C, 5% CO_2_ and 1% O_2_.

To model the AVF microenvironment, different combinations of direct co‐cultures of cells (100 000 cells/well) were used: NHF/HUVSMC, NHF/HUVEC, HUVSMC/HUVEC and NHF/HUVSMC/HUVEC. The same total number of cells was used for both these combinations and the mono‐cultures. For combinations of NHF/HUVSMC, NHF/HUVEC and HUVSMC/HUVEC, a ratio of 1 to 1 was used with 50% of each cell line. For NHF/HUVSMC/HUVEC, the ratio was still 1 to 1 but with 33% of each cell line.

### 2.2 Tissue collection

Studies on human samples received authorization from regulatory boards and the local ethics committee. Informed consent was obtained from patients regarding the collection of samples and data. Deidentified matured patent human AVF samples were provided by Yale Vascular Surgery. Samples were obtained after ≥6 months of haemodialysis during surgical revision of the fistula resulting from severe anastomotic stenosis.^45^ The samples analysed are of a segment of normally remodelling patent vein from the fistulae. Control veins were obtained from renal disease patients at the time of initial AVF creation. Sample procurement with informed consent was approved by Human Investigation Committee of Yale University Institutional Review Board HIC No. 1005006865. Tissues were fixed in formalin and embedded in paraffin.

### 2.3 Pharmacological inhibitors and chemicals

Recombinant human OPN was from R&D system and was used at 1 µg/mL. Inhibition of the NF‐κB activity was conducted with a specific inhibitor (inhibitor of IKK2 (AS602868), iNF‐κB), a gift from Dr JF Peyron.

### 2.4 RNAi transfection

Cells were plated at 70% confluence and transfected the following day using Lipofectamine RNAiMAx^TM^ (Thermo Fisher Scientific) according to the manufacturer's protocol, at a 50 nmol/L final concentration of RNAi. The set of RNAi sequences (Eurogentec) targeting human were as follows: SIMA (siCtl) (forward) 5′‐CCUACAUCCCGAUCGAUGAUG‐3′, siHIF‐1α (forward) 5′‐CUGAUGACCAGCAACUUGA‐3′,^46^, ^47^, ^48^ siHIF‐2α (forward) 5′‐CAGCAUCUUUGAUAGCAGU‐3′,^48^ siOPN#1 (forward) 5′‐GUUUCACAGCCACAAGGAC‐3′, siOPN#2 (forward) 5′‐GCCACAAGCAGUCCAGAUU‐3′.

### 2.5 Immunoblotting

Protein immunodetection was performed as previously ///described.^49^ Briefly, cells were lysed in 1.5X SDS buffer, and if exposed to hypoxia, they were lysed while inside the hypoxic chamber. Proteins (40 μg) were separated on 7.5% SDS‐polyacrylamide gels and transferred onto polyvinylidene difluoride membranes (Millipore). Blots were blocked in 5% milk in Tris‐HCl/NaCl buffer and incubated with antibodies at 4°C overnight. Immunoreactive signals were revealed with horseradish peroxidase (HRP)‐conjugated antibodies (Promega) using the Pierce^TM^ ECL Western blotting system (Thermo Fisher Scientific). The antibody against HIF‐1α was produced in our laboratory and used at 1:1000. The antibody against HIF‐2α (NB100‐122) was purchased from Novus Biologicals (Littleton) and used at 1:1000. The antibody against OPN (AF 14‐33OP) was purchased from R&D System and used at 1:500. The antibody against β‐tubulin (T4026; Sigma‐Aldrich) was used at a 1:2000 dilution as a loading control.

### 2.6 RNA extraction and quantitative real‐time PCR

For patient samples, total RNA was extracted with the RNeasy FFPE Kit (Qiagen). For cells, total RNA was extracted and reverse transcribed (SuperScript II Reverse Transcriptase; Invitrogen), and real‐time RT‐PCR was performed on an StepOnePlus Fast real‐time PCR system (SybR Green, Applied Biosystems) on triplicates as described. Results were normalized to the housekeeping gene *36B4* on the same plate. Differences in gene expression were calculated using the 2^−ΔΔ^ *^C^* ^t^ method.

Human primer sequences used were: *OPN* (Eurogentec) (forward: 5′‐CAGGCTGATTCTGGAAGTTCTGA‐3′; reverse: 5′‐GGGCTAGGAGATTCTGCTTCTGA‐3′), *GLUT1* (Eurogentec) (forward: 5′‐TTCACTGTCGTGTCGCTGTTT‐3′; reverse: 5′‐TCACACTTGGGAATCAGCCCC‐3′) and *rpLO* (Sigma) (forward: 5′‐ CAGCTTGGCTACCCAACTGTT‐3′; reverse: 5′‐GGCCAGGACTCGTTTGTACC‐3′).

### 2.7 ELISA

The concentration of human OPN was determined by ELISA (kit DuoSet ELISA Human osteopontin, R&D systems DY1433) using NHF, HUVSMC and HUVEC conditioned culture media according to the manufacturer's instructions. Cells were plated at 50% confluence and treated the following day with H_2_O_2_ (50 µmol/L), glucose (50 mmol/L) and inhibitor of IKK2 (AS602868), iNF‐κB^50^ for 48 hours. Treatment with phosphate‐buffered saline (PBS) was used as control.

### 2.8 Immunofluorescence

Human vein and AVF samples were sectioned at 5 μm and mounted onto glass slides. After heating overnight at 55°C, deparaffinization was accomplished through a series of xylene and graded ethanol soaks. Sections were heated in citric acid buffer (pH 6.0) at 100°C for 10 minutes for antigen retrieval, then washed and permeabilized with 0.1% Tween‐20 in phosphate‐buffered saline (PBS‐T). Antigens were blocked with 2% bovine serum albumin in PBS‐T for 60 minutes at room temperature. Sections were then incubated with the following primary antibodies diluted in antibody diluent (Agilent Dako S3022) overnight at 4°C: anti‐OPN (1:500, Abcam, ab8448), anti‐alpha‐smooth muscle actin (1 μg/mL, Invitrogen 14‐9760‐80). Sections were treated with secondary antibodies goat anti‐rabbit Alexa‐Fluor‐568 (Life Technologies) and chicken anti‐mouse Alexa‐Fluor‐488‐diluted 1:400 for 1 hour at room temperature. Sections were then stained with ProLong™ Gold Antifade Mountant with DAPI (Thermo Fisher), and coverslips were applied. Digital fluorescence images were captured with the EVOS FL Auto 2 cell imaging system (Thermo Fisher) at 40x, with subsequent image analysis done with ImageJ software (NIH). Osteopontin fluorescence was background‐subtracted and normalized to smooth muscle cell area (determined by area fraction of alpha‐smooth muscle cell actin immunofluorescence). The background was obtained from negative control sections, that is without the primary antibody. The value of this negative signal was subtracted from the signal obtained from the red channel (OPN) using the primary antibody. Samples were analysed with ImageJ, and whole sections were analysed; 3‐5 sections were analysed for each sample and the mean value computed for each specimen.

### 2.9 Statistics

All values are the means ± SEM. Statistical analyses were performed using Student's *t* test in Microsoft Excel. The *P* values are indicated. All categorical data used numbers and percentages. Quantitative data were presented using the median and range or mean. Differences between groups were evaluated using the chi‐square test for categorical variables and Student's *t* test for continuous variables. Analyses were performed using SPSS 16.0 statistical software (SPSS Inc.). All statistical tests were two‐sided, and *P*‐values < .05 indicated statistical significance whereas *P*‐values between .05 and .10 indicated a statistical tendency.

## 3 RESULTS

We suggested that OPN plays a role in intimal hyperplasia of AVF; thus, we searched in vitro for OPN expression in hypoxia (1% O_2_) in comparison to normoxia in cells (NHF, HUVSMC and HUVEC) representative of those involved in AVF maturation. We investigated silencing of HIFs and inhibition of NF‐κB *in vitro* in these cells to see if these transcription factors down‐regulated OPN expression in hypoxia.

### 3.1 Intracellular OPN (iOPN) is not induced in hypoxic cells

**Figure 1:**
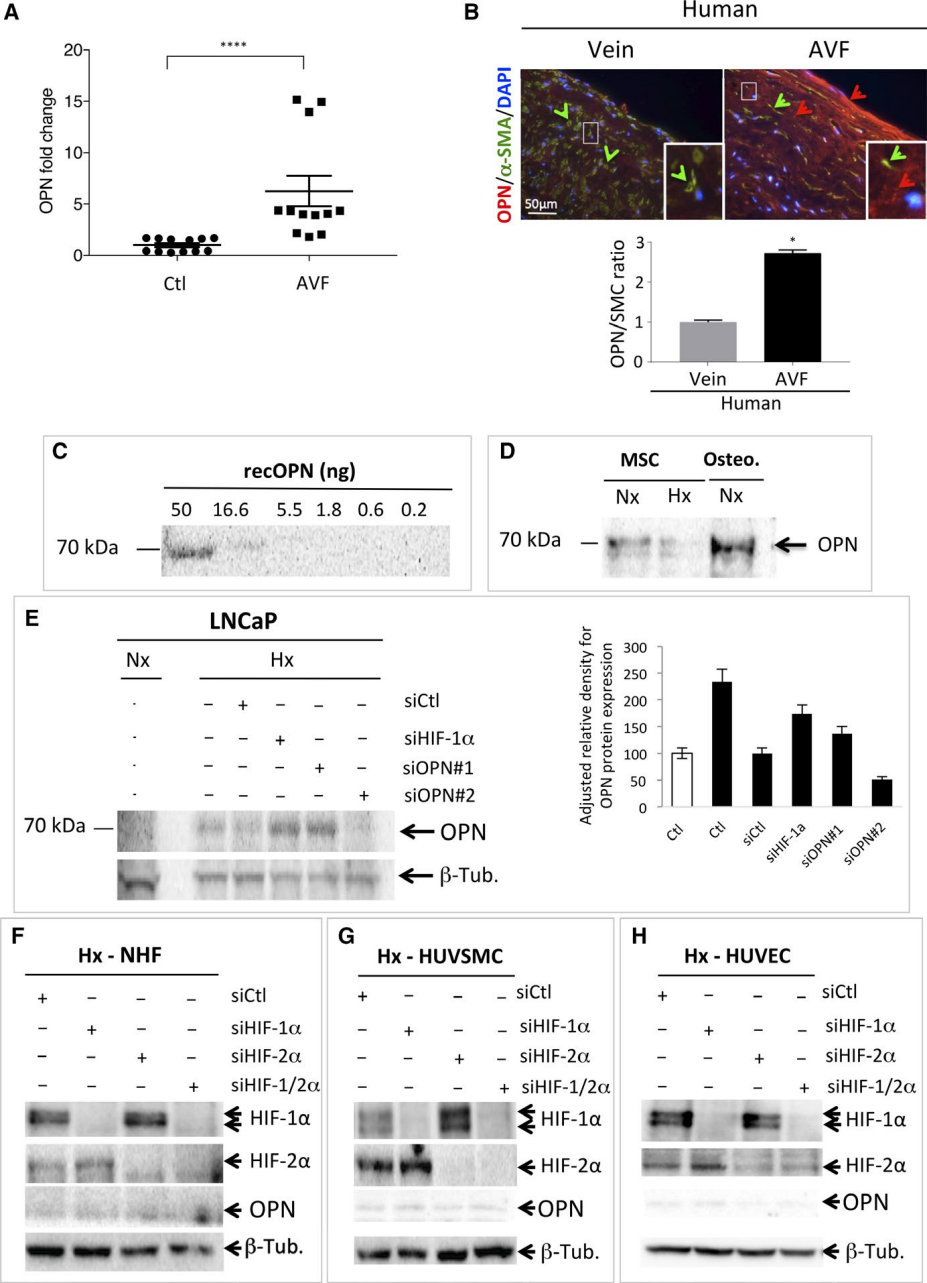
Osteopontin (OPN) is weakly expressed in NHF, HUVSMC and HUVEC and is not induced in hypoxia. A, Levels of OPN mRNA in AVF patients (n = 12) compared to control veins (Ctl). ****, *P* < .0001 (Test Mann‐Whitney). B, Representative immunofluorescence of OPN (red staining and arrow) and alpha‐smooth muscle actin (α‐SMA—green staining and arrows) of human control upper‐arm vein and venous limb of a mature AVF. Osteopontin quantification of group data (vein, n = 3; AVF, n = 2), unpaired *t* test, *P* < .01 (*right*). C, Quantitative immunoblotting to detect recombinant OPN protein. Serial dilutions from 50 to 0.2 ng were used. D, Bone marrow mesenchymal (MSC) and osteoblast (Osteo.) cell lysates analysed by immunoblotting for OPN as indicated. E, LNCap cells were transfected with control siRNA (siCtl), siHIF‐1α (40 nmol/L), siOPN#1 (40 nmol/L) and siOPN#2 (40 nmol/L) and incubated in normoxia (Nx) or hypoxia 1% O_2_ (Hx) for 48 h. Cell lysates were analysed by immunoblotting for OPN or β‐tubulin as indicated. Quantification of the signal to OPN is shown using ImageJ (OPN/β‐tubulin). F–H, NHF (F), HUVSMC (G) and HUVEC (H) cells were transfected with control siRNA (siCtl), siHIF‐1α (40 nmol/L), siHIF‐2α (40 nmol/L) and siHIF1/2α (40 nmol/L + 40 nmol/L), incubated in hypoxia 1% O_2_ (Hx) for 48 h. AVF, Arteriovenous fistula; HUVEC, human umbilical vein endothelial cells; HUVSMC, Human umbilical vein smooth muscle cells; NHF, normal human fibroblasts

First, we evaluated the level of expression of OPN in AVF patients. Both expressions of OPN mRNA (Figure 1A) and OPN immunoreactivity (Figure 1B) in the AVF were significantly increased, compared to control veins, confirming our previous results and the potential role of OPN in AVF maturation.^34^ Knowing that OPN has two isoforms, intracellular OPN (iOPN) and soluble OPN (sOPN), we first characterized iOPN by immunoblot in different cellular models. Recombinant OPN (recOPN) was used to quantitate the expression (Figure 1C). Human bone marrow mesenchymal cells osteoblasts (Figure 1D) and LNCap cells (Figure 1E) were examined as controls, since these two models are known to express OPN. Moreover, OPN is known as a biomarker for prostate cancer and for its role in tumour progression.^51^ The antibody against OPN detected recOPN at the concentrations of 50 and 16.6 ng and at a relative molecular weight of 70 kDa (Figure 1C). Human bone marrow mesenchymal cells and osteoblasts showed high expression of iOPN in normoxia after 48 hours incubation. Since iOPN is expressed in LNCaP, we evaluated potential hypoxic induction of iOPN in these cells and observed a slight induction (twofold; Figure 1E). As HIF‐1 is the key protein responsible for cellular adaptation to hypoxia, we checked whether HIF‐1 was involved in the induction of iOPN. Using siRNA to silence HIF‐1α, we showed that, in the absence of HIF‐1α, iOPN expression was still present. Only siRNA#2 to OPN completely abolished iOPN expression. Subsequent experiments were done with this siRNA to OPN. NHF (representing the adventitia; Figure 1F), HUVSMC (representing smooth muscle cells associated with elastic fibres) (Figure 1G) and HUVEC (representing the endothelium) (Figure 1H), the three cell lines that compose the three layers of the arteries and veins, were tested for iOPN expression. Although HIF‐1α and HIF‐2α were detected in hypoxic conditions, 1% O_2_ for 48 hours, none of these cells expressed iOPN. These cells did not express iOPN in either normoxia or hypoxia 0.2% O_2_ (data not shown). Transfection of NHF (Figure 1F), HUVSMC (Figure 1G) and HUVEC (Figure 1H) with siRNA to HIF‐1α and/or HIF‐2α strongly diminished the amount of these two subunits and the silencing of HIF‐1α slightly increased the detection level of HIF‐2α and *vice versa*. Thus, we silenced both subunits to completely abolish the HIF activity.

These results suggest that iOPN expression is below the detectable level of 16.6 ng, (Figure 1C) in NHF, and weakly expressed in HUVSMC and HUVEC.

### 3.2 Secreted OPN (sOPN) is not induced in hypoxic cells

**Figure 2:**
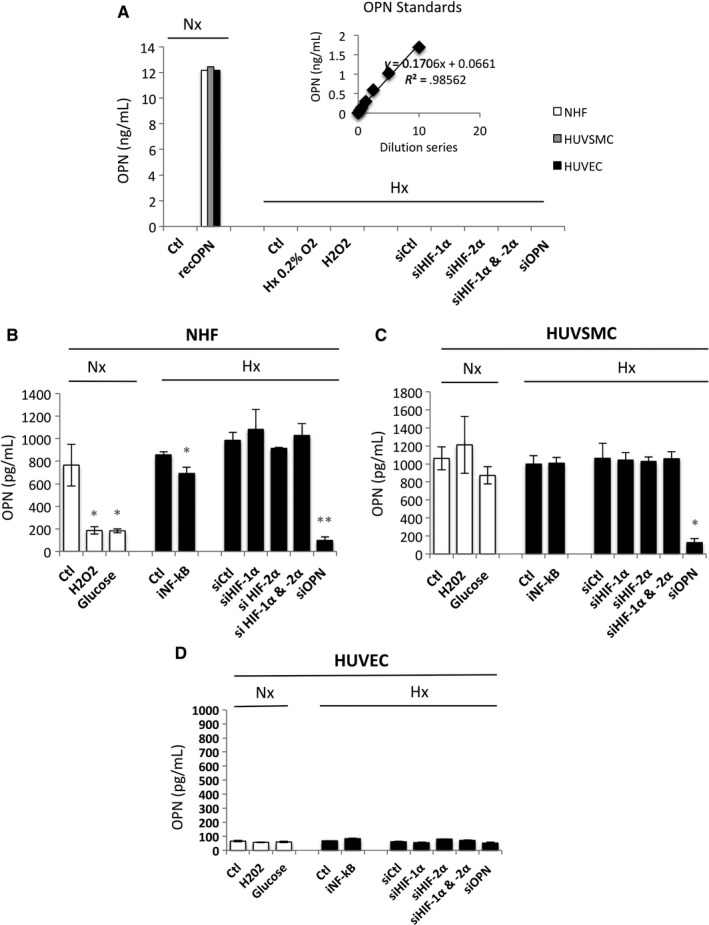
Production of OPN by NHF, HUVSMC and HUVEC in normoxia compared to hypoxia. (A) Histograms representing the level of OPN produced *in vitro* by NHF, HUVSMC and HUVEC after 48 h in culture, detected by ELISA using a Quantikine ELISA Human Osteopontin immunoassay. The mean ± SEM is representative of three independent experiments expressed as nanograms per millilitre. Cells were cultured in the absence (Ctl) or presence of recombinant OPN (recOPN) in normoxia. Cells were cultured in the absence (Hx 0.2% O_2_) or presence of H_2_O_2_ (50 µmol/L) for 48 h or transfected with control siRNA (siCtl), siHIF‐1α (40 nmol/L), siHIF‐2α (40 nmol/L), siHIF‐1/2α (40 nmol/L + 40 nmol/L) or siOPN (40 nmol/L), and incubated in hypoxia 0.1% O_2_ (Hx). (B–D) Histograms representing the level of OPN produced in vitro by NHF (B), HUVSMC (C) and HUVEC (D) after 48 h in culture using a DuoSet ELISA Human osteopontin immunoassay. The mean ± SEM is representative of three independent experiments expressed as picograms per millilitre. Cells were cultured in the absence (Ctl) or presence of H_2_O_2_ (50 µmol/L), glucose (50 mmol/L) for 48 h, or transfected with control siRNA (siCtl) with addition of glucose (Gluc) + siOPN (40 nmol/L) in normoxia. Cells were cultured in the absence (Ctl) or presence of an inhibitor to NF‐kB (iNF‐kB) (10 µmol/L) for 48 h or transfected with control siRNA (siCtl), siHIF‐1α (40 nmol/L), siHIF‐2α (40 nmol/L), siHIF‐1/2α (40 nmol/L + 40 nmol/L) or siOPN (40 nmol/L), and incubated in hypoxia 1% O_2_ (Hx) for 48 h. The mean ± SEM is representative of three independent experiments carried out in triplicate. HUVEC, human umbilical vein endothelial cells; HUVSMC, Human umbilical vein smooth muscle cells; NHF, normal human fibroblasts; OPN, osteopontin

We then tested for sOPN in these cells using an ELISA. Two different kits were used, one that measured sOPN in nanograms (Figure 2A) and one in picograms (Figure 2B). No sOPN was detected in NHF, HUVSMC and HUVEC, at nanogram levels when using recOPN as reference, in normoxia or hypoxia or in conditions that are supposed to induce OPN such as H_2_O_2_ or glucose^52^, ^53^ (Figure 2A). No cell death was observed in these conditions (data not shown). A more sensitive kit detected sOPN in NHF (Figure 2B), HUVSMC (Figure 2C) but not in HUVEC (Figure 2D). HNF and HUVSMC showed a similar level of sOPN in normoxia, 765 and 1060 pg/mL, respectively. SiRNA to OPN (siOPN), used as a control, abolished secretion of OPN. However, no stimuli known to increase OPN, including H_2_O_2_ or glucose, activated sOPN in normoxia. Hypoxic conditions did not increase sOPN and silencing of HIF‐1α and/or HIF‐2α did not block secretion of OPN in NHF and HUVSMC. However, inhibition of NF‐κB activity with a specific inhibitor (inhibitor of IKK2 (AS602868), iNF‐κB) slightly, but statistically, decreased secretion of OPN from 850 to 700 pg/mL.

These results confirm weak expression of both iOPN (Figure 1) and sOPN in these cells and show that sOPN was not detected in HUVEC. They also confirm that these two forms are not induced under hypoxic conditions *via* HIFs. However, they show that NF‐κB participates in OPN regulation to some extent.

### 3.3 mRNA expression of OPN is not induced in hypoxic cells

**Figure 3:**
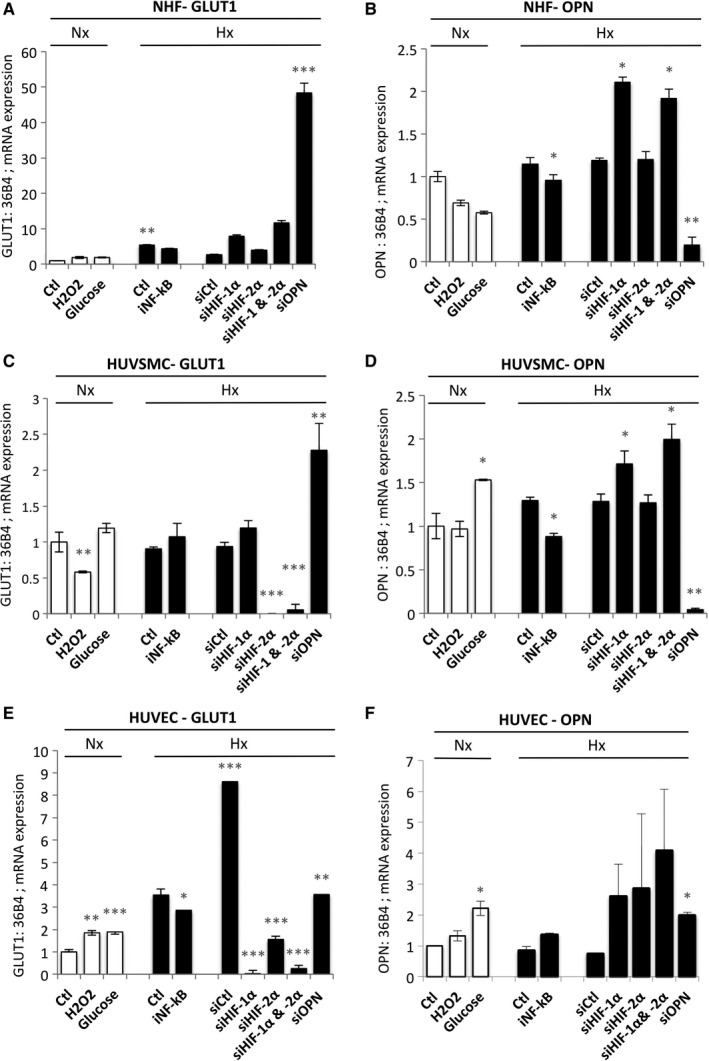
Expression of OPN mRNA in NHF, HUVSMC and HUVEC is not induced in hypoxia. (A, C and E) Histograms represent the expression of the mRNA of GLUT‐1 in NHF (A), HUVSMC (C) and HUVEC (E). The mean ± SEM is representative of three independent experiments. Cells were cultured in the absence (Ctl) or presence of H_2_O_2_ (50 µmol/L), or glucose (50 mmol/L) for 48 h, or transfected with control siRNA (siCtl) and glucose (Gluc) + siOPN (40 nmol/L) in normoxia. Cells were cultured in the absence (Ctl) or presence of an inhibitor to (iNF‐kB) (10 µmol/L) for 48 h or transfected with control siRNA (siCtl), siHIF‐1α (40 nmol/L), siHIF‐2α (40 nmol/L), siHIF‐1/2α (40 nmol/L + 40 nmol/L) or siOPN (40 nmol/L), and incubated in hypoxia 1% O_2_ (Hx) for 48 h. (B, D and F) Histograms represent the expression of the mRNA of OPN in NHF (B), HUVSMC (D) and HUVEC (F). The mean ± SEM is representative of three independent experiments. Cells were cultured in the absence (Ctl) or presence of H_2_O_2_ (50 µmol/L), glucose (50 mmol/L) for 48 h, or transfected with control siRNA (siCtl) and glucose (Gluc) + siOPN (40 nmol/L) in normoxia. Cells were cultured in the absence (Ctl) or presence of iNF‐kB (10 µmol/L) for 48 h or transfected with control siRNA (siCtl), siHIF‐1α (40 nmol/L), siHIF‐2α (40 nmol/L), siHIF‐1/2α (40 nmol/L + 40 nmol/L) or siOPN (40 nmol/L), and incubated in hypoxia 1% O_2_ (Hx) for 48 h. HUVEC, human umbilical vein endothelial cells; HUVSMC, Human umbilical vein smooth muscle cells; NHF, normal human fibroblasts; OPN, osteopontin

As we were unable to detect hypoxic induction of either iOPN or sOPN, we tested an even more sensitive technique to measure OPN. Using real‐time qPCR, we quantified the endogenous expression of OPN in normoxia and hypoxia (Figure 3). As a control for hypoxia, the glucose transporter 1 (GLUT1) mRNA expression was evaluated (Figure 3A,3,3). Two‐ to threefold induction at the mRNA level of GLUT1 was observed in NHF (Figure 3A) and HUVEC (Figure 3E). A decrease in GLUT1 expression was observed with the HIF‐2α ‐directed siRNA in NHF and HUVSMC, whereas both siRNAs to HIF‐1α and/or HIF‐2α decreased GLUT1 expression in HUVEC suggesting shared regulation by HIF‐1 and HIF‐2 in these cells. SiRNA to HIF‐1α and HIF‐2α abolished endogenous expression of both HIF‐1α and HIF‐2α as shown in Figure S1. Intriguingly, the mRNA level of GLUT1 was systematically increased when OPN was silenced, suggesting negative control of GLUT1 by OPN. Thus, we quantified the endogenous expression of OPN in NHF (Figure 3B), HUVSMC (Figure 3D) and HUVEC (Figure 3F). For the first time, we observed an increase of OPN mRNA levels in the presence of glucose in HUVSMC and HUVEC but not in NHF. However, no induction under conditions of hypoxia was detected in NHF, HUVSMC and HUVEC. A slight decrease in OPN expression was observed in NHF and HUVSMC in the presence of iNF‐κB confirming that NF‐κB participates in OPN regulation. A twofold induction was observed at the mRNA level for OPN in hypoxia in NHF with siHIF‐1α and siHIF‐1/2α compared to the control siRNA suggesting that HIF‐1α only could repress OPN. Similarly, a threefold induction was observed at the mRNA level for OPN in hypoxia in HUVEC with siHIF‐1α, but also with siHIF‐2α and siHIF‐1/2α. However, siRNA to OPN did not affect the mRNA level of OPN under these conditions; therefore, these results (Figure 3F) are not specific to OPN expression and reflect non‐specific amplification due to undetectable OPN expression in HUVEC (Figure 2D).

We thus confirmed that mRNA expression of OPN is not induced in hypoxia in the three cell lines tested.

### 3.4 Co‐culture favours OPN induction in hypoxia

**Figure 4:**
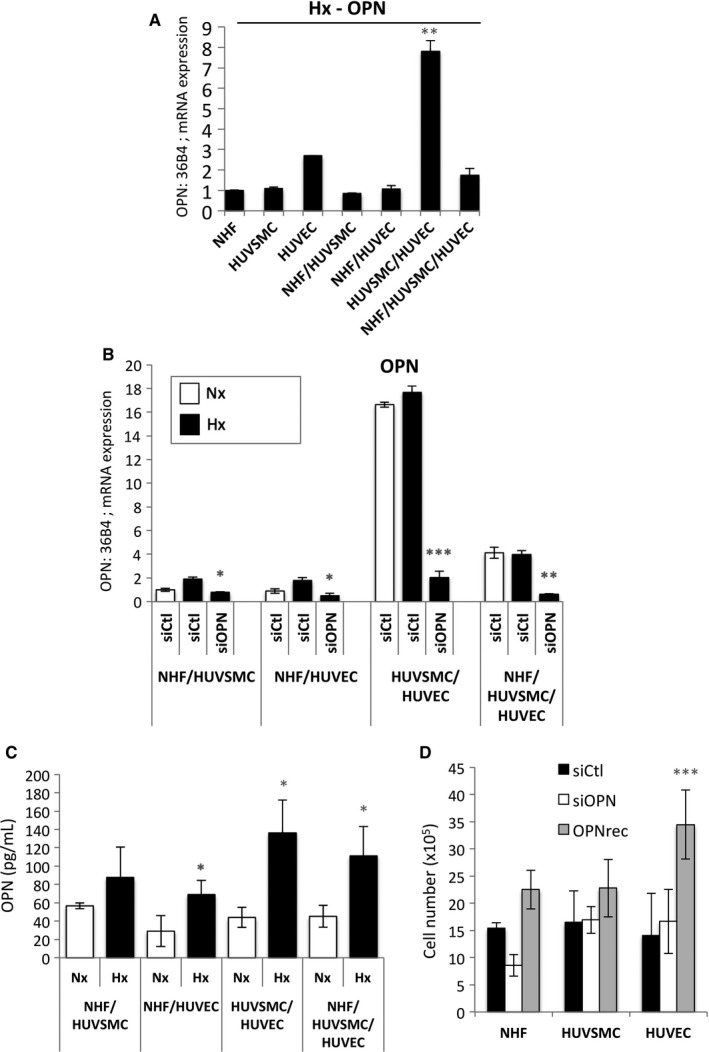
Co‐culture induced OPN expression in hypoxia. (A) Histograms represent the expression of the mRNA of OPN in mono‐ and co‐cultures after 48 h in hypoxia 1% O_2_ (Hx). The mean ± SEM is representative of three independent experiments. (B) Histograms represent the expression of the mRNA of OPN in co‐culture (mean ± SD of values from three independent experiments). Co‐culture with NHF/HUVSMC, NHF/HUVEC, HUVSMC/HUVEC, NHF/HUVSMC/HUVEC were transfected with control siRNA (siCtl) in normoxia (Nx) or transfected with control siRNA (siCtl) or siOPN (40 nmol/L), and incubated in hypoxia 1% O_2_ (Hx) for 48 h. (C) Histograms represent the level of OPN produced in vitro in co‐culture of NHF/HUVSMC, NHF/HUVEC, HUVSMC/HUVEC and NHF/HUVSMC/HUVEC, after 48 h in culture detected with a DuoSet ELISA Human osteopontin immunoassay. The mean ± SEM is representative of three independent experiments expressed as picograms per millilitre. (D) NHF‐, HUVSMC‐ and HUVEc cells were transfected with control siRNA (siCtl) in normoxia (Nx) or transfected with siOPN (40 nmol/L) or in the presence of recombinant human OPN (OPNrec—1 µg/mL) and incubated in normoxia for 48 h. Cell lines were seeded at the same density and incubated in for 48 h. The mean ± SEM is representative of two independent experiments carried out in duplicate. HUVEC, human umbilical vein endothelial cells; HUVSMC, Human umbilical vein smooth muscle cells; NHF, normal human fibroblasts; OPN, osteopontin

Finally, given that a potential link between OPN and AVF has been shown early in the maturation process in a mouse model where the three layers of cells are juxtaposed, we co‐cultured cells using different combinations: NHF/HUVSMC, NHF/HUVEC, HUVSMC/HUVEC and NHF/HUVSMC/HUVEC (Figure 4). The mRNA expression of OPN in NHF, HUVSMC and HUVEC was higher than that of NHF/HUVSMC, NHF/HUVEC, HUVSMC/HUVEC and NHF/HUVSMC/HUVEC co‐cultures (Figure 4A). Co‐culture of HUVSMC/HUVEC clearly increased the level of OPN expression. We then quantified the endogenous expression of OPN in normoxia and hypoxia in the absence or presence of siRNA compared to control siRNA or siOPN (Figure 4B). Co‐cultures of NHF/HUVSMC and NHF/HUVEC exposed to hypoxia showed a twofold increase in OPN expression. Cell‐cell interaction between HUVSMC and HUVEC showed high basal expression of OPN, 16‐fold induction, compared to the other co‐cultures in which no induction was found in hypoxia. Finally, all the cell types together presented intermediate expression, fourfold induction in normoxia, but still no induction in hypoxia.

Therefore, we measured sOPN in the different combinations using ELISA (Figure 4C). For the first time, sOPN induction was observed in all co‐culture combinations in hypoxia. Moreover, the presence of HUVEC in combination with NHF (2.3‐fold induction), HUVSMC (2.7‐fold induction) or NHF/HUVSMC (2.4‐fold induction) seemed to stimulate more sOPN induction in hypoxia compared to the combination without HUVEC (NHF/HUVSMC, 1.5‐fold induction). Finally, we showed that OPN down‐regulation did not alter cell proliferation of NHF, HUVSMC and HUVEC, whereas recombinant human OPN only increased HUVEC proliferation (Figure 4D) suggesting that HUVEC cells were sensitive to OPN and could directly contribute to the pathological NH.

These results strongly suggest that in the absence of cell‐cell interaction, OPN expression is not induced in hypoxia. Moreover, HUVEC cells, which do not express OPN, are central to the process of NH.

## 4 DISCUSSION

The main objective of this study was to determine whether OPN was induced in hypoxia and thus could be responsible for driving the failure of AVF maturation due to NH and juxtaanastomotic stenosis. We first used cell mono‐layers grown on tissue culture plastic to examine hypoxic production of OPN observed in AVF, which is the approach used by the majority of research groups. Mono‐cultures are less complex to perform but certainly less adapted to, and not very representative of, the physiological extracellular microenvironment present in humans. While the expression of OPN has been shown to be increased during early AVF maturation^54^ or throughout maturation^34^ under hypoxic conditions,^44^ none of the three cell lines forming the three layers of the artery and vein walls expressed iOPN or sOPN.

However, this is the first time that a study has shown that cell‐cell communication or interaction between NHF, HUVSMC and HUVEC is essential for OPN induction in hypoxia. Co‐culture allows investigation of cell‐cell interactions and is therefore more representative of human in vivo‐like tissue models.^55^ Interestingly, all co‐cultures showed induction of OPN in hypoxia, whereas none of the mono‐cultures showed OPN induction. However, we noted that co‐cultures containing HUVEC produced more OPN. Although we do not know which cell line(s) produced the OPN, it is interesting to note that the amount of sOPN in mono‐culture conditions was barely detectable and 10 times lower in HUVEC than in NHF or HUVSMC. Moreover, siRNA directed to OPN in NHF and HUVSMC totally abolished OPN at the mRNA level, whereas it had no effect on HUVEC suggesting that the mRNA level of OPN was extremely low in HUVEC. However, OPN was only induced in the presence of HUVEC in the co‐culture. Moreover, HUVEC cells were the most sensitive to the presence of OPN as it increased their proliferation. However, were the HUVEC cells the only ones producing OPN? If this is the case, the regulation of OPN production in HUVEC may be a consequence of specific cellular interactions and regulation. Hall et al^34^ showed a 40‐fold increase in OPN expression in the endothelium of veins of AVF relative to control veins suggesting OPN production was mainly in the intima. In this murine AVF model, they also showed increased expression of matrix metalloproteinases (MMPs, MMP3 and MMP9) early on after AVF creation and subsequent decreased expression after 21 days. They proposed that such a balance could favour AVF failure and stenosis. When OPN was co‐localized with members of the MMP family, it was shown to induce the expression of MMP9,^56^ one of the two MMPs identified by Hall et al^34^ In our model, hypoxic induction of OPN, an active player in tissue remodelling and a response to vascular injury and inflammation, may modulate the activity of MMPs, which are responsible for degradation and accumulation of the ECM, and thus contribute to AVF failure and NH.

In conclusion, changes in oxygenation that occur during AVF maturation may up‐regulate secretion of OPN, possibly *via* endothelial cells or at least through cell‐cell interactions of the different cell layers that form AVF, and in turn increase inflammation and promote endothelial cell proliferation.

## CONFLICT OF INTEREST

The authors declare that they have no conflicting interests.

## AUTHOR CONTRIBUTIONS

N.S, J.C, AD and NMM: conception and design, data analysis and interpretation, manuscript writing and editing; J.C, M.D, Ju.P., LF: data collection and assembly; M.R and JFP: provided reagents and cells, data analysis and interpretation; R.H‐K, Ja.P., FB E.J‐B: data analysis and interpretation, manuscript editing and final approval of manuscript.

## Data Availability

I confirm that my article contains a Data Availability Statement even if no data are available (list of sample statements) unless my article type does not require one (eg, Editorials, Corrections, Book Reviews, etc). I confirm that I have included a citation for available data in my references section, unless my article type is exempt.
