# Supplementary figures and images for: Co‐culture of human fibroblasts, smooth muscle and endothelial cells promotes osteopontin induction in hypoxia
Source: J Cell Mol Med. 2020 Feb 7;24(5):2931–41. doi: 10.1111/jcmm.14905 (PMC7077551; doi:10.1111/jcmm.14905)

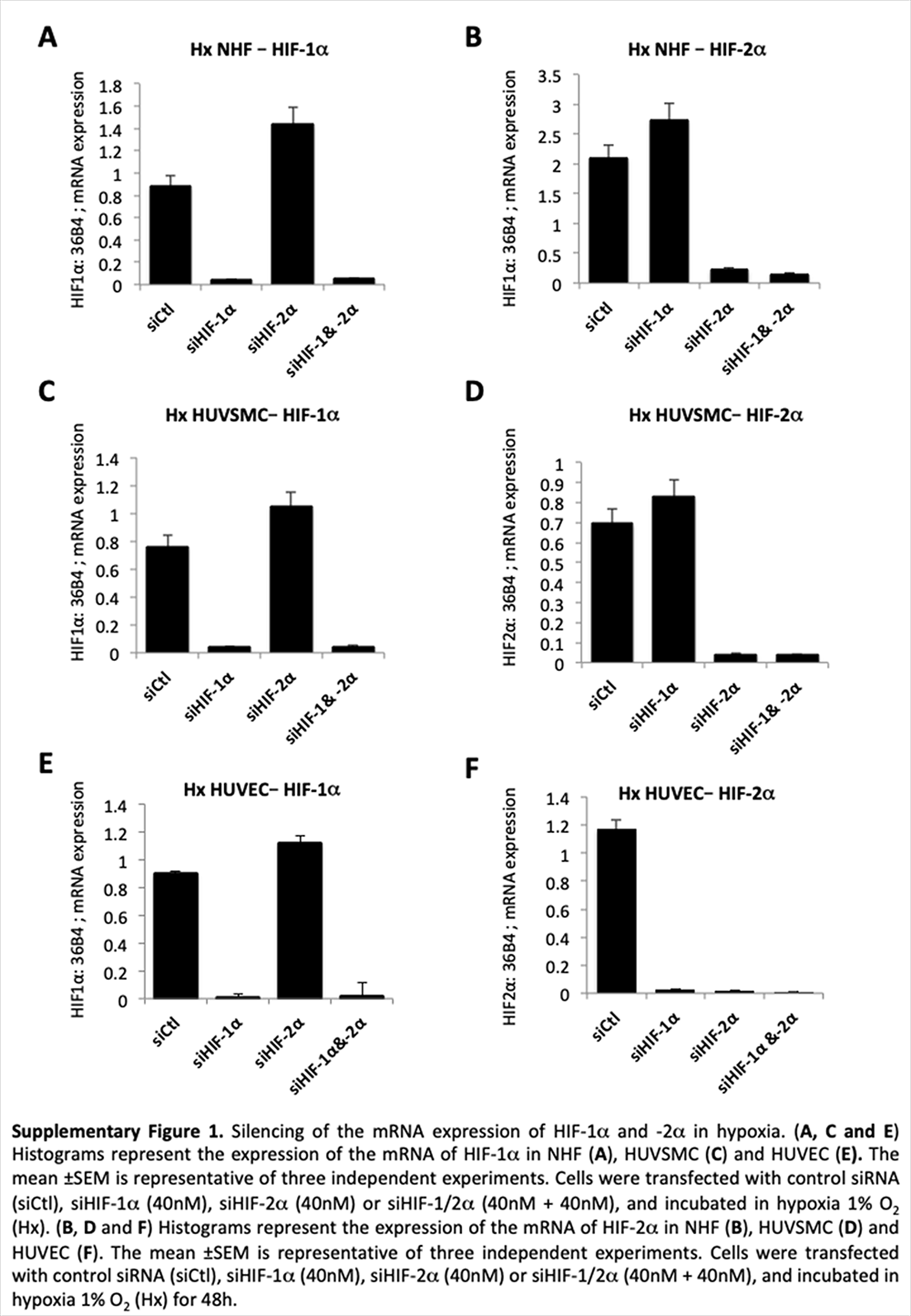

Supplement: Supplementary file 1 [file JCMM-24-2931-s001.tiff]
